# Functional outcome and fusion rates of translaminar screw fixation of the lumbar and lumbosacral spine: A case series

**DOI:** 10.1016/j.ijscr.2022.106906

**Published:** 2022-03-01

**Authors:** Andhika Yudistira, Maulana Hasymi Hutabarat, Lasa Dhakka Siahaan, Muhammad Alwy Sugiarto

**Affiliations:** Orthopaedic and Traumatology Department, Faculty of Medicine Universitas Brawijaya, RSUD Dr. Saiful Anwar, Malang, Indonesia

**Keywords:** Translaminar screw, Fusion rate, ODI score, Posterior lumbar fusion

## Abstract

**Introduction:**

Pedicle screw fixation has been the gold standard because this technique allows solid arthrodesis and provides a degree of stiffness that immediately diminishes mechanical back pain. Although the translaminar screw technique is considered inferior to the pedicle screw technique due to the thought that it is minimally invasive, studies proved that both fixation systems had significantly greater stiffness and reduced range of motion compared with the normal vertebrae. The purpose of this study is to determine the fusion rate, the clinical outcome of translaminar screw fixation of the lumbar and lumbosacral for the long term.

**Case presentation:**

We evaluate six patients with a degenerative lumbar disorder and performed posterior lumbar fixation and fusion with the translaminar screw. The translaminar screw was performed monosegmentally (3 patients), across two segments (1 patient), and across three segments (2 patients). We then evaluate Oswestry Disability Index (ODI) score one week, three months, and one year postoperatively.

**Discussion:**

When lumbosacral spine fusion procedures are performed without supplemental internal fixation, a 10% pseudoarthrosis rate can be expected for single-level fusions, and the percentage can reach 30% if more than two levels are fused. Compared to the pedicle screw technique, the translaminar technique provides a limited profile and less bony invasion that will minimize the risk of failure.

**Conclusion:**

Translaminar screw offers immediate postoperative stability, seen in postoperatively, patients experienced better quality of life than preoperatively. It also represents a useful and inexpensive technique for short segment fusion of the non-traumatic lumbar and lumbosacral spine.

## Highlights

• Subjective assessment showed all patients experienced symptoms relieved post-surgery.
• The translaminar technique provides less bony invasion to minimize the risk of failure.
• The biomechanical test reveals translaminar screw increased the stiffness 2.4 times.
• This technique can be an alternative for fusions in the degenerative lumbar disease.

## 1 Introduction

Magerl first introduced the translaminar screw as a modification from the transfacet technique described by King in 1948 [1], [2], [3]. Although he reported a 100% fusion rate in a single-level fusion, King's technique carried a risk of injury to the nerve root due to the placement of the screw (near the foramen and the nerve root). Magerl's technique of inserting screws from the contralateral side through the lamina and ended at the base of the transverse process lowers this risk [4]. His technique was superior in terms of more bony aspect, can be done under direct visual control, and parallel to the direction of exiting root.

Since the late 1980s, pedicle screw fixation has been the gold standard because this technique allows solid arthrodesis and provides a degree of stiffness that immediately diminishes mechanical back pain. However, its safety, insertion facility, and morbidity have been clearly and consistently reported in the literature. In addition, this technique also jeopardizes the paraspinal muscle due to traction and has been shown to injure these muscles functionally and increase the volume of devitalized tissue, leading to an increased incidence of infection with additional complications such as dural tears, neurological injury, and an increased rate of infection due to long operating time [5]. Another issue is that the most cephalad pedicle screws of a pedicle screw plate or rod build can induce mechanical impairment of the facet joint above the fused levels if not applied with caution [1], [3], [6].

Although the translaminar screw technique is considered inferior to the pedicle screw technique due to the thought that it is minimally invasive, one study conducted by Ferrara et al. proved that the translaminar screw technique was found to be equivalent to bilateral pedicle screw placement [3], [7]. Both fixation systems had significantly greater stiffness and reduced range of motion compared with the normal vertebrae [7].

The purpose of this study is to determine the fusion rate, the clinical outcome of translaminar screw fixation of the lumbar and lumbosacral for the long term. The author report six cases of posterior instrumentation using translaminar screw technique followed up with subjective assessment using ODI (Oswestry Disability Score) preoperatively and postoperatively, and objective assessment using radiographic evaluation [8]. This report has followed PROCESS checklist and guidelines, and consent was given to our patient regarding the data obtained in this case would be submitted for publication [9].

## 2 Case presentation

**Fig. 1:**
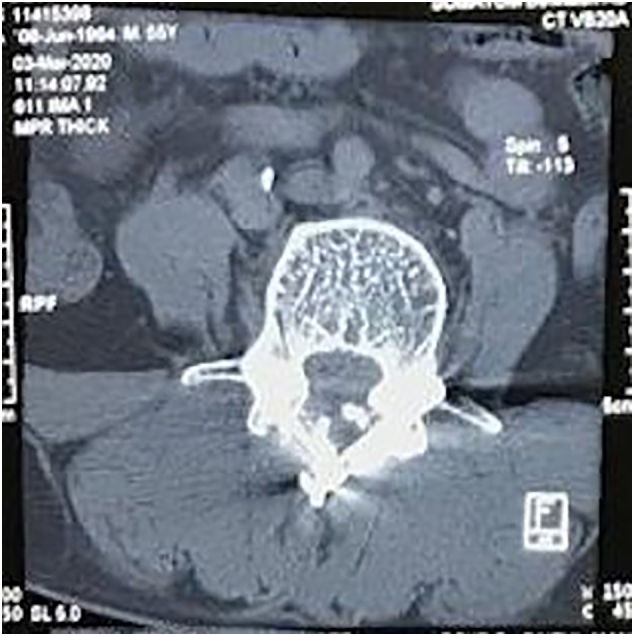
Computed tomography scan of a translaminar screw fixation eight months after surgery. The screws are ideally placed, crossing the facet joint perpendicularly and ending in the base of the transverse process.

We present six cases of degenerative lumbar disorder with a history of back pain more than one year that underwent posterior stabilization using the translaminar screw technique described by Magerl (Fig. 1, Fig. 5). This is prospective study in single center with translaminar screw fixation cases in two years. All the translaminar screw fixation performed in academic tertiary hospital in Indonesia by experienced orthopaedic surgeon. Fixation and fusion with the translaminar screw were performed monosegmentally in three patients, across two segments in one patient and across three segments in two patients using facet screw (partially threaded) with size 4.5 × 40 mm. All the patients were wearing orthoses for three months after the surgery to prevent excessive loads on the implants, and all patients were followed up for 12 months and assessed radiologically using plain X-ray, clinically using ODI score.

**Fig. 2:**
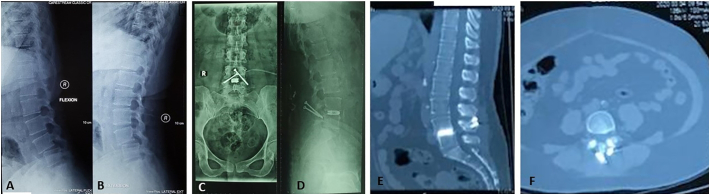
A 58-year-old female with low back pain for more than one year. Radiological examination reveals spondylolisthesis on level L4-L5 (A, B). The patient underwent the translaminar screw fixation and showed no sign of pain. X-ray and CT Scan show union on level L4-L5 (C–F).

The radiologic investigation was done by anteroposterior, flexion-extension, and lateral x-ray film to detect segmental motion, 12 months postoperative. A solid bony fusion with a radiologically calcified fusion mass, no apparent motion on the flexion-extension x-rays in the fused segment was documented in all patients, there were no Perioperative or late infections, and there was no neurologic injury observed (Fig. 2).

**Fig. 3:**
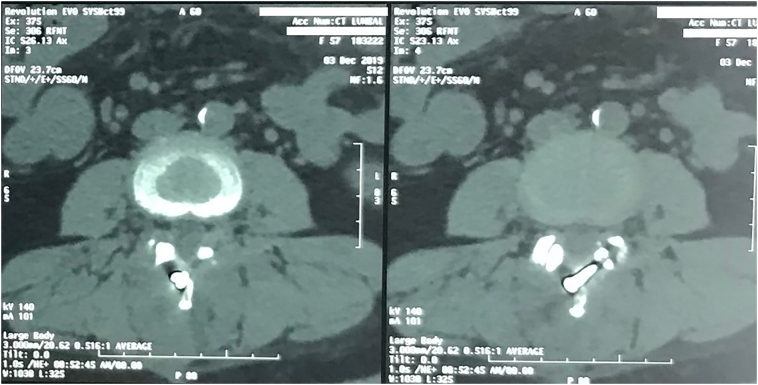
CT Scan of a 58-year-old female one year after surgery. There was a radiolucent halo, indicating peri-implant osteolysis around the screw.

**Fig. 4:**
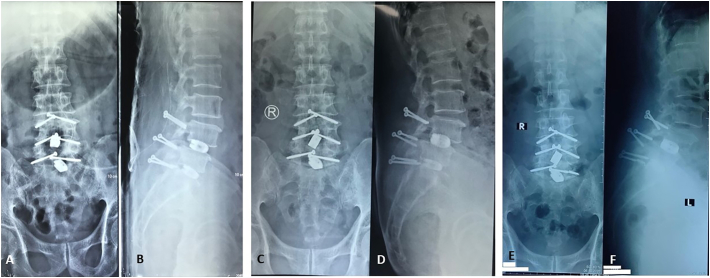
A 56-year-old male with chronic low back pain. Translaminar screw fixation was performed in 3 segments (A, B). X-ray shows progressing spondylolisthesis at L4-L5 (C, D) and broken screw at L5-S1 (E, F).

Loosening of the screw (radiolucent area) was noted in one patient (fixation across three segments). However, no segmental motion could be detected on the flexion-extension radiographs. The screw fragments remained without causing any symptoms, and no migration was detected. The radiolucent halo surrounding the implant was found in one patient. This line was measured, and the length was more than 1 mm, which was considered positive for osteolysis. This sign indicates instability on the site of arthrodesis (Fig. 3). However, there was no progressive spondylolisthesis, and subjective assessment showed improvement of quality of life. Implant failure was confirmed in 1 patient, which had three segments fixated. Spondylolisthesis was seen two months after surgery, and the broken screw was found one year after surgery (Fig. 4). There was no history of trauma after surgery, no neurologic deficit, no segmental motion on the flexion-extension radiographs; the screw fragments did not cause any symptoms, and no migration was reported.

**Fig. 5:**
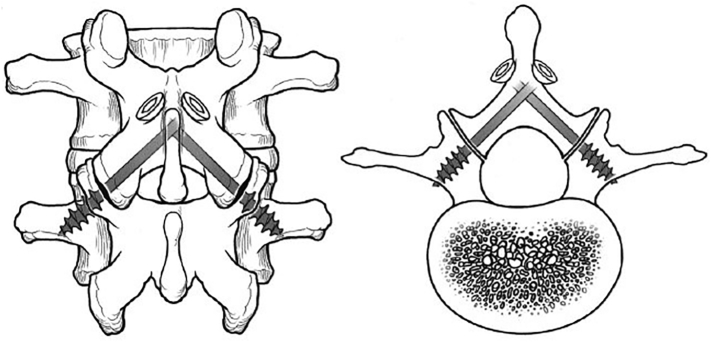
Translaminar screw fixation. The facet joints are diarthrodial joints and are the only true articulations in the lumbosacral spine, thus directly fixating through the facet joints appears logical for stabilization of the spine.

Each patient completed a questionnaire to evaluate subjective results using the ODI score (Table 1). Based on the ODI score, five patients were categorized as crippled (83%), and one patient was categorized as bed-bound (17%). These patients had back pain which impinged on all aspects of patients' life. All patients were followed for one month and 12 months postoperative. We found score improvement in all patients during the first month, and improvement was found 12 months postoperative (Table 2).

**Table 1:** Interpretation of ODI score.

ODI Score	Interpretation
0–20%	The patient can cope with most living activities. Usually, no treatment is indicated apart from advice on lifting sitting and exercise.
21%–40%	The patient experiences more pain and difficulty with sitting, lifting and standing. Travel and social life are more difficult and they may be disabled from work. Personal care, sexual activity and sleeping are not grossly affected and the patient can usually be managed by conservative means.
41%–60%	Pain remains the main problem in this group but activities of daily living are affected. These patients require a detailed investigation.
61%–80%	Back pain impinges on all aspects of the patient's life. Positive intervention is required.
81%–100%	These patients are either bed-bound or exaggerating their symptoms.

**Table 2:** Oswestry disability index score.

No.	Identity	ODI Score (Pre-operative)	ODI Score (1 month postoperative	ODI Score (12 months postoperative)
1.	Female, 58 y.o	71,1%	53,3%	33,3%
2.	Male, 55 y.o	82,2%	24,4%	15,5%
3.	Male, 69 y.o	80%	28,8%	15,5%
4.	Female, 58 y.o	66,6%	0	0
5.	Female, 55 y.o	80%	2,2%	0
6.	Female, 63 y.o	80%	53,3%	44,4%

## 3 Discussion

When lumbosacral spine fusion procedures are performed without supplemental internal fixation, a pseudoarthrosis rate of about 10% can be expected for single-level fusions, and the percentage can reach 30% if more than two or three levels are fused [1], [5]. The goal of translaminar screw fixation is to immobilize facet joints and augment fusion between adjacent vertebra [2]. After performing decompression and stabilization using the translaminar screw technique, we evaluate the success rate using the ODI score. Subjective assessment using ODI score showed that all patients experienced symptoms relieved, progressively one month until 12 months after surgery. This indicates the achievement of one goal in spine surgery, which is pain-free.

Five fundamental requirements will influence fusion: (1) availability of an adequate population of osteogenic cells, (2) the presence of an osteoconductive matrix within the region where new bone tissue is desired, (3) osteoinductive signals within the graft site, (4) adequate local blood supply to support a bone healing response, and (5) local mechanical environment suitable for bone formation [7]. The technique can be achieved with the pedicle screw technique or translaminar screw fixation. In biomechanical study of the role of supplementary translaminar screw fixation discovered that additional posterior translaminar screw fixation provided significant stability in flexion and lateral bending modes while reducing motion in extension and axial rotation in the stand-alone anterior lumbar interbody fusion with BAK cages [5]. furthermore, compared to the pedicle screw technique, the translaminar technique provides a limited profile and less bony invasion to minimize the risk of failure [4]. And in a biomechanical study showed that the translaminar screw technique would increase stiffness in flexion 30% higher than the pedicle screw technique [7].

Fusion procedures are usually augmented with instrumentation to minimize the motion across an interbody bone graft, thus hopefully resulting in an increased fusion rate [1], [10]. However, rigid fixation can have detrimental effects if the fixation does not allow adequate stresses and micromotion to be transmitted to the bone graft. Excessive rigidity of the spinal implant can cause stress shielding. This inhibits the bone graft's ability to experience stresses incurred with daily spinal loading, thus resulting in resorption of the graft. Conversely, excessive motion across a bone graft can contribute to pseudarthrosis and early instrumentation failure [7].

Heggeness tested the translaminar screw fixation Biomechanically in his study, using seven fresh cadaver spines with static eccentric loading and cyclic loading using servohydraulic material testing machine. The static eccentric loading test reveals that after instrumentation with translaminar screw fixation, the stiffness increased 2.4 times compared to the uninstrumented spines. Cyclic loading reveals that after 5000 cycles, the stiffness did not decrease significantly, concluding that the translaminar screw fixation can maintain the increased stiffness, thus improving the fusion rate [1].

In this case series, solid bony fusion and no apparent motion on the flexion-extension x-rays in the fused segment were documented in all patients. However, loosening of the screw was noted in one patient. However, no segmental motion could be detected on the flexion-extension radiographs. Heggeness in his study also reported that one case of loosening of the screw resulted in pseudoarthrosis, but the back pain in that case resolved in one year [1]. This complication was also found in markwalder's study, he found one case from 74 cases with loosening of the screw without pseudoarthrosis, and the patient is asymptomatic [11].

Implant failure was confirmed in one patient, which had three segments fixated despite there was no history of trauma after surgery. However, we found no neurologic deficit. Implant failure was also reported in Humke's study; He reported two from 173 cases who have implant failure on monosegmental fusion. Both patients did not show segmental motion on the flexion-extension radiographs, the screw fragments did not cause any symptoms, and no migration was reported [6].

In this study, there was no evidence of neurologic injury. According to other researchers who have described the same procedures, severe neurologic complications also not documented in conjunction with translaminar screws of the lumbar spine [1], [6], [11]. The screws travel tangentially to the spinal canal along the lamina, reducing the chance of perforating the ventral laminar cortex. As a result, this surgery for internal spinal fixation can be regarded safe and technically simple [6].

The minimal volume of screws may lower the possible danger of infection compared to transpedicular devices. This also leaves plenty of room for graft material to be placed, promoting firm bone fusion. Translaminar screws also use lower-cost implants compared to transpedicular screws [6]. Markwalder also said that the translaminar screw fixation procedure is noteworthy because it provides posterior internal fixation of the lumbar spine and the lumbosacral joint monosegmental stabilization with anatomical spinal canal reconstruction, enlargement of the intervertebral foramina, and preservation of the posterior bony aspects of the osseous neural arch [11].

The use of postoperative external immobilization is important to provide fusion with the correct position and minimalized the risk of implant failure and nonunion due to excessive loads on the implants, primarily in translaminar screw fixation [10]. In this case series the external immobilization were used by the patient three months postoperatively, other studies also suggesting three months is the optimal time for external immobilization postoperatively [6], [10].

Some experts believe that the risk of applying pedicle screw systems for degenerative lumbar and lumbosacral spine fusion is insufficient because of major anterior deficiency of the spine. The translaminar screw technique relies on an anatomically intact anterior column that can resist compression force. This is the reason why such a technique is ideal for degenerative cases presented in this study [6].

This study adds information that translaminar screw fixation can be an alternative to posterior stabilization, especially in degenerative lumbar disease. The limitation of this study is a single-center retrospective study and did not prospectively compare with other approaches, the size of the patient included in this study is small. Therefore, we came with case series, and the relevant data is sufficient to describe the functional outcome and fusion rates of translaminar screw fixation. For future studies, we recommend using a larger size sample or patients with more evaluation scoring and biomechanical test, if possible, compared to transpedicular screw fixation.

## 4 Conclusion

The enhancement of lumbar and lumbosacral fusion with the translaminar screw technique offers an easy way to improve quality of life, fusion rates and simplify postoperative management. This technique can be an alternative, useful, inexpensive and safe method for short segment fusions in patients with non-traumatic lumbar and lumbosacral spine problems.

## Funding

This research did not receive any specific grant from funding agencies in the public, commercial, or not-for-profit sectors.

## Consent

Written informed consent was obtained from the patient for publication of this case report and accompanying images. A copy of the written consent is available for review by the Editor-in-Chief of this journal on request.

## Provenance and peer review

Not commissioned, externally peer-reviewed.

## Ethical approval

This study has been reviewed and approved by the authors' Institutional Review Board.

## Registration of research studies

This Case report is not “First in Man” Study.

## Guarantor

Andhika Yudistira.

## CRediT authorship contribution statement

Andhika Yudistira: conceptualization, writing original draft preparation, supervision, project administrator, validation.

Maulana Hasymi Hutabarat: data collecting, data interpretation, writing original draft preparation, writing the paper and editing, validation.

Lasa Dhakka Siahaan: data collecting, data interpretation, writing original draft preparation, writing the paper and editing, validation.

Muhammad Alwy Sugiarto: data collecting, data interpretation, writing original draft preparation, writing the paper and editing, validation.

## Declaration of competing interest

We declare that they have no known competing financial interests or personal relationships that could have appeared to influence the work reported in this paper.
